# Intensive Hemodiafiltration Successfully Removes Ganciclovir Overdose and Largely Exceeds Reported Elimination During Hemodialysis—A Case Report and Review of the Literature

**DOI:** 10.3389/fphar.2020.00882

**Published:** 2020-06-12

**Authors:** Verena Gotta, Anne Leuppi-Taegtmeyer, Mirjam Gessler, Marc Pfister, Daniel Müller, Andreas Werner Jehle

**Affiliations:** ^1^Pediatric Pharmacology and Pharmacometrics, University of Basel Children’s Hospital, Basel, Switzerland; ^2^Division of Clinical Pharmacology and Toxicology, University Hospital Basel, Basel, Switzerland; ^3^National Poisons Information Centre, Tox Info Suisse, Associated Institute of the University of Zurich, Zurich, Switzerland; ^4^Institute of Clinical Chemistry, University Hospital Zürich, Zurich, Switzerland; ^5^Department of Internal Medicine, Hirslanden Klinik St. Anna, Lucerne, Switzerland; ^6^Transplantation Immunology and Nephrology, University Hospital Basel, Basel, Switzerland

**Keywords:** ganciclovir, valganciclovir, hemodialysis, hemodiafiltration, overdose, toxicity, blood-to-plasma concentration ratio

## Abstract

We present the case of a kidney transplant patient (Cockroft-Gault estimated creatinine clearance 14 ml/min) who was inadvertently eight-fold overdosed with a single dose of 500 mg intravenous ganciclovir. To prevent the immunosuppressed patient from being exposed to severe risks of prolonged ganciclovir overdosing, including potentially fatal bone marrow suppression and severe neurotoxicity, the patient was treated with hemodiafiltration (HDF) to enhance drug elimination. Since the product label reports a 50% decrease of ganciclovir plasma concentrations after intermittent hemodialysis (HD), two HDF sessions were considered necessary to achieve a ≥75% elimination of the drug by precaution, despite targeted intense HDF prescription. Ganciclovir plasma concentration data were obtained during both HDF sessions and were analyzed retrospectively. Pharmacokinetic analysis revealed that prescribed HDF successfully decreased drug plasma concentrations by ≥90%. This ganciclovir reduction ratio matched the urea reduction ratio achieved (≥92%). Model-based assessment of ganciclovir dialysis clearance (estimated to be 445 ml/min), accounting for its two-compartmental kinetics, was higher than urea dialysis clearance (estimated to be 310 ml/min). This suggests potential relevant accumulation of ganciclovir into blood cells, at least in this patient after overdosing. The amount (fraction) of drug removed by 1^st^ HDF was estimated to 269 mg (93% of total amount of 288 mg eliminated during the 1^st^ HDF session; estimated amount in the body prior to 1^st^ HDF: 380 mg). A literature review was performed to summarize and systematically compare available information on ganciclovir elimination during intermittent renal replacement therapy. In conclusion, the high ganciclovir HDF clearance measured in our patient largely exceeded previously reported elimination during HD, meaning that HDF prescription was highly efficient in the present case, and that a second HDF session might not have been necessary. This finding may be considered to guide renal replacement therapy in the scope of drug overdosing. It may also be evaluated for ganciclovir dose adjustment in patients on chronic HD or HDF with high small solute clearance, since a strong correlation between ganciclovir and urea elimination efficiency was observed.

## Background

Active cytomegalovirus (CMV) infection and disease is a major and common complication in solid organ transplant recipients and is associated with an increased risk of all types of infections, graft loss, and with decreased survival. Intravenous ganciclovir is an antiviral agent of choice for prevention and treatment of CMV infections in this population (Kotton et al., 2018). It is a nucleoside analogue that requires intracellular activation by phosphorylation *via* viral and cellular kinases. The active triphosphate derivative acts as an inhibitor of viral DNA polymerases and shows a long intra-cellular half-life (~16.5h) compared to plasma half-life of the unphosphorylated parent drug (2–4 h in patients with normal renal function) (Scott et al., 2004).

Ganciclovir is a small molecule (molecular weight 255.2 Da) with low protein binding (2%) and a small volume of distribution (0.54–0.87 l/kg). It is mainly (>90%) cleared renally by glomerular filtration and active tubular secretion (Cymevene Swiss Product Information., ). Hence, dose adjustment in renal impairment is necessary, with the elimination half-life of ganciclovir being prolonged up to >10-fold in patients with renal failure (30 h in patients with endstage renal disease) (Lake et al., 1988). Pharmacological properties suggest efficient elimination by hemodialysis (HD) (diffusive dialysis clearance) and hemodiafiltration (HDF) (by both diffusive and convective clearance) (Gotta et al., 2017; Gotta et al., 2020). The manufacturer states that one HD session decreases ganciclovir plasma concentrations by approximately 50% and suggests considering HD to treat overdosing. Indeed, cases of fatal bone marrow suppression have been reported in patients with renal failure who were accidentally overdosed due to omission of dose adjustment according to renal function (Ar et al., 2009). Under therapeutic doses, neutropenia is a common side effect occurring in approximately one third of patients, however in this setting, it is mostly reversible. Further severe adverse reactions associated with overdose may include neurotoxicity (confusion, hallucinations, seizures, coma), acute renal failure, and hepatitis (Cymevene Swiss Product Information., ).

We present a case of a kidney transplant patient with an estimated creatinine clearance of 14 ml/min (Cockroft-Gault) who accidentally received an eight-fold overdose with a single dose of 500 mg ganciclovir and who was treated with HDF to enhance drug elimination. According to available literature two HDF sessions were performed to ensure an 75% elimination of the drug. Ganciclovir plasma concentration data obtained during HDF and analyzed retrospectively revealed that the first HDF session successfully decreased drug plasma concentration by 92%, suggesting that a second HDF session might not have been necessary.

We report our case according to the EQUATOR (Enhancing the QUAlity and Transparency Of health Research) network recommended guidelines for case studies on extracorporeal treatments in poisonings (Lavergne et al., 2014).

## Case Presentation

A 70-year old woman, who received a deceased donor renal transplant in 2017, was treated with ganciclovir for CMV disease with ulcerative esophagitis. In this patient weighing 44 kg with an estimated creatinine clearance of 14 ml/min ganciclovir was started at a reduced dose of 65 mg i.v. 22 months after transplantation. At this time the patient was under immunosuppressive therapy with tacrolimus and prednisolone, whereas mycophenolate mofetil was paused due to intermitted neutropenia. Eleven days after start of ganciclovir treatment, the patient received inadvertently an overdose of ganciclovir with a single dose of 500 mg.

To prevent the immunosuppressed patient from being exposed to severe risks of prolonged ganciclovir overdosing, a first session of intermittent post-dilution HDF with standard blood and dialysate flow (**Table 1**) and high ultrafiltration (34 L per 4 h session) was started 5 h after recognizing the dosing error. Using a dialysate containing 4 mmol/L potassium and additional administration of potassium phosphate (30 mmol over 4 h) successfully prevented hypokalemia and hypophosphatemia despite highly intense HDF.

**Table 1:** Patient and hemodiafiltration characteristics.

Patient characteristics	
Gender	Female
Age	70 years
Body weight	44 kg
Serum creatinine (pre-dialysis)	237 µmol/L
Estimated CL_crea_ (Cockroft-Gault)	14 ml/min
Hematocrit	27%
Serum albumin	24.1 g/L
Comedication	tacrolimus, prednisolone, sulfamethoxazole/trimethoprim (prophylaxis), high-dose pantoprazole, chondroitin sulfate/hyaluronic acid, unfractionated heparin (subcutaneous prophylaxis), low-dose acetylsalicylic acid, nebivolol, citalopram, low-dose quetiapine
**Hemodiafiltration parameters**^1^	
Filter type	FX CorDiax 100, Fresenius (2.2 m^2^, KoA_urea_: 1545 ml/min (BC Renal Network, 2019))
Blood flow (Q_B_)	350 ml/min (21 L/h)
Dialysate flow (QD)	525 ml/min (31 L/h)
Calculated ultrafiltration rate (postdilution)	131 ml/min (7.9 L/h, or 34.8 L, 1^st^ session) and 118 ml/min (7.1 L/h, or 40.1 L, 2^nd^ session)
Session duration	265 min (4.42 h, 1^st^ session) and 285 min (4.75 h, 2^nd^ session)
**Achieved urea (reference) clearance**	
Urea (pre- and post dialysis)	1^st^ session: 21 and 1.6 mmol/L 2^nd^ session: 2.2 and <0.5 mmol/L
Corresponding urea reduction ratio (URR)	1^st^ session: 92% 2^nd^ session: >97%
Corresponding delivered spKt/V according to Daugirdas JT 1993 (Daugirdas, 1993) (reported by dialyzer)	1^st^ session: 3.2 (reported: 2.41) 2^nd^ session: >1.6 (reported: 2.74)
Predicted urea clearance from Q_B_, Q_D_ and filter KoA_urea_	230 ml/min (13.8 L/h)^5^ = 79% of *in vitro* clearance of 292 ml/min (Michaels, 1966) (17.6 L/h) (Michaels, 1966)

^1^The aggressive therapy was only possible with potassium at 4.1 mmol/l in dialysate and additional administration of 30 mmol potassium phosphate during first hemodiafiltration. During the 2^nd^ session dialysate with 5.1 mmol/l potassium was used. KoA urea, mass-transfer area coefficient of urea; URR, urea reduction ratio calculated as: (C_pre_ − C_post_)/C_pre_ × 100%.

A complete summary of patient characteristics, concomitant drug treatments, dialysis prescription, and efficiency in terms of small molecule clearance (measured by urea clearance) is given in **Table 1**.

Written informed consent was obtained from the patient for publication of her case.

### Pharmacokinetic Sampling and Calculated Kinetic Parameters

**Figure 1:**
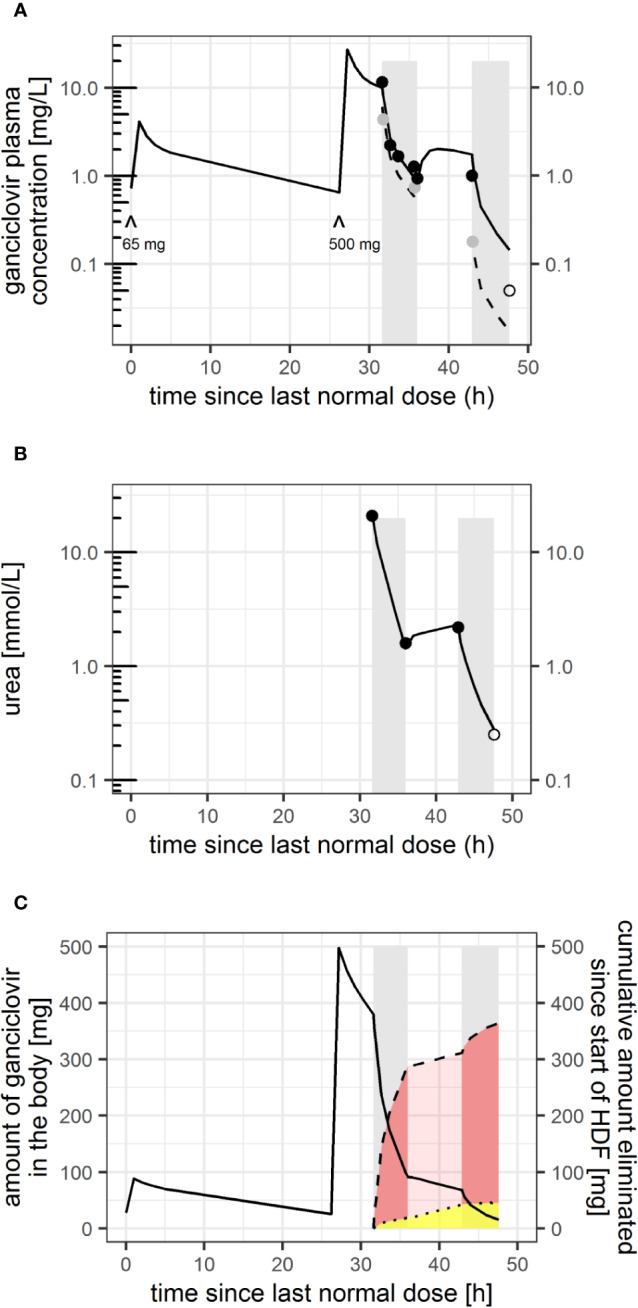
**(A)** Ganciclovir plasma concentration measured (*black dots*: pre-filter, *gray dots*: post-filter) and model-predicted individual concentration-time profile since last normal (for renal function adjusted) ganciclovir dose (*black line*: pre-filter and intra-dialytic plasma concentration, *dashed line*: post-filter concentration assuming a constant extraction ratio within each hemodiafiltration session). ^: time and dose of i.v. ganciclovir administered. *Gray bars* indicate time on hemodiafiltration. **(B)** Urea concentration measured (*dots*) and model-predicted urea concentration-time profile. *Open circles*: (pre-filter) concentrations below limit of quantification (LOQ) (0.1 for ganciclovir, 0.5 mmol/L for urea), plotted at LOQ/2. **(C)** model-predicted amount of ganciclovir in the body (black solid line), and predicted total amount eliminated since start of first HDF session (dashed line), which is the sum of elimination by residual ganciclovir clearance of the patient (yellow shaded area, limited by dotted line) and HDF (red shaded area).

Plasma samples for pharmacokinetic analysis were taken before, during and after the two HDF sessions at in total eight time points. Paired pre- and post-filter concentration samples (C_pre-filter_ and C_post-filter_) were taken at three time points to calculate the extraction ratio (ER) (**Table 2A**, **Figure 1A**):

$$\mathrm{ER}=\left(C_{\mathrm{pre}-\mathrm{filter}}-C_{\mathrm{post}-\mathrm{filter}}\right)/C_{\mathrm{pre}-\mathrm{filter}}$$

**Table 2:** **(A)** Ganciclovir plasma concentrations measured during 2 dialysis sessions and calculated extraction ratio (ER) and reduction ratio (RR). **(B)** Predicted RR from model simulations under different alternative scenarios.

*(A) Ganciclovir measurements and calculated extraction ratio (ER) and reduction ratio (RR)*				
Session	Time (h)	C_pre-filter/_C_post-filter_ (mg/L)	Calculated ER	Calculated RR
1	Start = 0	11.59/4.38 (+2 min)	0.622	
1	1	2.23/*Not measured*	–	
1	2	1.67/*Not measured*	–	
1	4	1.27/0.74 (+2 min)	0.417	
1	Stop = 4.42	0.94/*Not measured*	–	92%
2	Start = 11.25	1.01/0.18 (+5 min)	0.822	
2	Stop = 16	<0.1/*Not measured*	–	>90%
			Mean: 0.62 SD: ± 0.20	
***(B) Model simulations: predicted reduction ratio (RR)***				
**Scenario**	**(a) CL_R,off_ [ml/min]** → predicted RR%	**(b) HD duration [h]** → predicted RR	**(c) CL_D_ [ml/min]** → predicted RR	**Total predicted RR** (scenario a+b+c)
1 = present case	**29** → 93%	**4.4** → 93%	445 → 93%	**93%**
2	**15** → 92%	**4.0** → 92%	**315 (−30%) →** 89%	**87%**
3	**0** → 92%	**3.5** → 90%	**100 (−80%) →** 64%	**51%**

The CL_D_ value in scenario 2 corresponds to a ≈30% reduced clearance expectation in the absence of high ultrafiltration (according to urea kinetic modeling). The CL_D_ value in scenario 3 corresponds approximately to the highest CL_D_ value reported in the literature under chronic hemodialysis. Note that also different distribution parameters may change RR, those were however assumed to be unchanged in model simulations.

ER, extraction ratio calculated as: (C_pre-filter_ – C_post-filter_)/C_pre-filter_; RR, ganciclovir reduction ratio calculated as: (C_start_ − C_stop_)/C_start_ × 100%; SD, standard deviation; C_pre-filter_, ganciclovir plasma concentration measured from sample drawn before the filter; C_post-filter_, ganciclovir plasma concentration measured from sample drawn after the filter.

The ganciclovir reduction ratio (RR) during HDF was calculated in line with the calculated urea reduction ratio (**Table 1**) from pre-HDF (C_start_, at start of HDF) and post-HDF (C_end_, after stopping HDF) ganciclovir pre-filter concentrations:

$$\mathrm{RR}=\left(C_{\mathrm{start}}-C_{\mathrm{end}}\right)/C_{\mathrm{start}}\times 100\%$$

Calculated ganciclovir HDF clearance (K_HDF_) was derived from expected diffusive (Kd) and convective clearance (Kc), accounting for the average ER measured:

$$\mathrm{Kd}=Q_{B}\times R_{\mathrm{bp}}\times \mathrm{ER}=350\mathrm{ml}/\min\times 1\times 0.62=217\mathrm{ml}/\min$$

Where Q_B_ = prescribed blood flow, R_bp_ = blood-to-plasma concentration ratio (assumed to equal 1 as average from *in silico* (Lukacova et al., 2016) and *in vitro* (Perrottet et al., 2007) estimates), and ER = average extraction ratio of 0.62.

Kc=UF×SR×f=118-131ml/min×1×0.38 = 45−50ml/min

With UF = ultrafiltration used (131 ml/min for 1^st^ session and 118 ml/min for 2^nd^ session, respectively), SR = solute ratio (or sieving coefficient), assumed to equal 1 for a small molecule like ganciclovir, and f = factor by which Kc is expected to be reduced in the presence of diffusive clearance (=(1-Kd/Qb) in this calculation (Depner, 2012))

Total calculated K_HDF_ hence calculates to:

$$K_{\mathrm{HDF}}=\;\mathrm{Kd}+\mathrm{Kc}=262-267\mathrm{ml}/\min$$

Note that uncertainties exist concerning Rbp – in literature on ganciclovir dialysis clearance (see below) no distribution into blood cells has partly been assumed (i.e. Rbp = 1-hematocrit, which would result in a lower Kd estimate for our patient of 217 ml/min × (1–0.27) = 158 ml/min, and K_HDF_ of 203–208 ml/min), while pharmacologically distribution into blood cells is necessary for its mechanism of action.

Concentrations were measured by liquid chromatography coupled to tandem mass spectrometry (LC-MS/MS) using an accredited and validated method. Imprecision of the method expressed as coefficient of variation was <7.3%, cumulative trueness from the last four rounds of external quality assessment (EQA) was −5.2%. EQA samples were obtained from KKGT (Den Haag, The Netherlands).

### Model-Based Estimation of Ganciclovir HDF Clearance and Fraction of Drug Removed by HDF

Due to uncertainties in K_HDF_ calculation mentioned above, and uncertainties in the volume of distribution to be used for calculating the amount and fraction of drug removed by HDF (A_removed,HDF_ and f_removed,HDF_, respectively), especially for this drug which is known to have two-comparmental distribution kinetics in plasma, a model-based pharmacokinetic analysis was performed using NONMEM (version 7.4.1, Icon Development Solutions, Ellicott City, MD) to estimate individual ganciclovir renal and HDF clearances (CL_R_ and CL_HDF_), and to derive A_removed,HDF_ and f_removed,HDF_. Typical pharmacokinetic parameters of a two-compartmental model were used according to the population pharmacokinetic model published by Caldes et al. developed from a population of solid organ transplant patients with mean body weight of 66 kg (Caldés et al., 2009) (mixed residual error: proportional error: 14.3%, additive error: ± 0.465 mg/L). The last concentration measured below the limit of quantification (LOQ) of 0.1 mg/L was set to LOQ/2.

In the used model the prior expectation (typical value) of renal clearance (CL_R_) is a function of estimated creatinine clearance (CL_crea_ − Cockroft-Gault estimate) (Caldés et al., 2009):

$$\mathrm{prior}\;CL_{R}\left(L/h\right)=7.49L/h\times \left(CL_{\mathrm{crea}}\left(\mathrm{ml}/\min\right)/57\mathrm{ml}/\min\right)=7.49\times \left(14/57\right)L/h=1.83L/h$$

Peripheral distribution parameters (peripheral volume of distribution, V_2_, and inter-compartmental distribution clearance Q) were additionally allometrically scaled by body weight, since physiologically those kinetic parameters are expected to be lower in a 44 kg patient as compared to a 66 kg patient for a molecule that mainly distributes within total body water:

$$V_{2}\left(L\right)=32.0\times \mathrm{weight}/66=21.3L$$

$$Q\left(L/h\right)=10.2\times {\left(\mathrm{weight}/66\right)}^{0.75}=7.52L/h$$

Total clearance (CL) was set equal to the sum of CL_R_ and CL_HDF_ during hemodialysis and to CL_R_ off-dialysis:

$$\mathrm{CL}=CL_{R}+CL_{\mathrm{HDF}}=CL_{\mathrm{tot},\mathrm{on}}\text{ during analysis}$$

$$\mathrm{CL}=CL_{R}=CL_{R,\mathrm{off}}\text{ off-dialysis}$$

Based on those parameters, expected inter-patient variability (33% in prior CL_R_, 48% in central volume of distribution, V_1_, with prior [typical] value of V_1_ = 31.9 L (Caldés et al., 2009)), and the measured pre-filter ganciclovir concentration samples, individual parameters of CL_R_, V_1_, and CL_HDF_ were estimated using Bayesian feedback to the following maximum a posteriori estimates: CL_R_ = 1.8 (95% CI: 1.29–2.60) L/h [= 30 ml/min], V_1_ = 14.1 (95% CI: 7.4–26.8) L, and CL_HDF_ = 26.7 L/h (relative standard error <1%) [445 ml/min]. Inter-session variability in CL_HDF_ was evaluated and was—with an estimate of <1%—considered negligible. Based on individual distribution and clearance parameters, the amount of ganciclovir in the body prior to the first HDF session was predicted to be 380 mg, which was reduced by 288 mg (76%) during the first HDF session. 269 mg (93%) were predicted to be eliminated by HDF (=A_removed,HDF_ and f_removed,HDF_) and 19 mg (7%) by patient’s residual clearance (**Figure 1C**).

In a sensitivity analysis, CL_HDF_ was fixed to calculatory K_HDF_, which yielded individual estimates of CL_R_= 2.4 L/h [40 ml/min] and V_1_ = 9.9 L, and a slightly worse model fit (P = 0.02, likelihood ratio test). The amount of ganciclovir in the body prior to the first HDF session was predicted to 320 mg, which was reduced by 220 mg (69%) during the first HDF session. 191 mg (89%) were predicted to be eliminated by HDF (=A_removed,HDF_ and f_removed,HDF_) and 29 mg (13%) by patient’s residual clearance.

### Modeling of Post-Filter Ganciclovir Concentrations

Post-filter concentrations (C_post-filter_) were modeled assuming a constant ER, to estimate the effective kinetic plasma flow through the filter (Q_EFF_) (Atkinson and Umans, 2009):

$$C_{\mathrm{post}-\mathrm{filter}}=C_{\mathrm{pre}-\mathrm{filter}}\times \left(1-\mathrm{ER}\right)=C_{\mathrm{pre}-\mathrm{filter}}\times \left(1-CL_{\mathrm{HDF}}/Q_{\mathrm{EFF}}\right)$$

Assuming diffusive clearance to represent the major mechanism of elimination for a small molecule during HDF (Tattersall and Blankestijn, 2019), Q_EFF_ would be limited by the blood flow (Q_B_) through the filter, but can exceed Q_B_ for molecules that accumulate in blood cells, i.e. for drugs with a blood-to-plasma concentration ratio (R_bp_) >1, since Q_EFF_ = Q_B_ × R_bp_ (Atkinson and Umans, 2009). For molecules that do not distribute into blood cells Q_EFF_ would in turn equal the plasma water flow, i.e. Q_EFF_ = Q_B_ × (1 − hematocrit). In HDF, Q_EFF_ can further increase by high ultrafiltration.

Allowing for inter-session variability in the ER (suggested in **Table 2**), Q_EFF_ was estimated to be 47.4 L/h (relative standard error <1%) [788 ml/min], corresponding to a mean ER of 0.56, with considerable inter-session variability of 49%. The proportional residual error in predicted C_post-filter_ concentrations was estimated to be 25%, an additive error was not quantifiable.

Modelling C_post-filter_ did not affect the ganciclovir CL_HDF_ estimate.

### Ganciclovir Model Simulations

Model simulations using different hypothetical CL_R_, dialysis duration, and dialysis clearance were performed using Berkeley Madonna (version 8.3.18) to evaluate the impact of those parameters on the reduction ratio (**Table 2B**), and to better compare our results to literature (see below).

### Comparative Kinetic Analysis of Urea as Reference Solute

Urea—just like ganciclovir—follows two-compartmental distribution kinetics (Clark et al., 1999). Urea dialysis clearance (CL_HDF,urea_) was also estimated kinetically using a model-based analysis. Urea distribution parameters (V_1_, V_2_, Q) and residual error were fixed to values predicted from a urea kinetic model accounting for weight-based changes in inter-compartmental clearance (Gotta et al., 2020). Renal urea clearance (CL_R,urea_) was assumed to be related to creatinine clearance (CL_R,crea_ as follows:

$$CL_{R,\mathrm{urea}}=CL_{R,\mathrm{crea}}\times 0.85/1.15=10.3\mathrm{ml}/\min$$

considering their correlation with glomerular filtration rate (GRF), which is approximately 15% overpredicted by CL_R,crea_ (undergoing tubular secretion) and 15% underpredicted by CL_R,urea_ (Almond et al., 2008).

The urea generation rate was initially fixed to 0.104 mmol/min (Clark et al., 1999). In a second step an individual value was estimated using prior values estimated from a small population of patients on chronic HD (prior mean: 0.17 mmol/min, inter-individual variability: 38%) (Pfister et al., 2004). The last concentration measured below the LOQ of 0.5 mmol/L was set to LOQ/2.

Total urea clearance (CL_urea_) was set equal to the sum of CL_R,urea_ and CL_HDF,urea_ during hemodialysis and to CL_R,urea_ off-dialysis:

$$CL_{\mathrm{urea}}=CL_{R,\mathrm{urea}}+CL_{\mathrm{HDF},\mathrm{urea}}=CL_{\mathrm{tot},\mathrm{urea},\mathrm{on}}\text{ during dialysis}$$

$$CL_{\mathrm{urea}}=CL_{R,\mathrm{urea}}=CL_{R,\mathrm{urea},\mathrm{off}}\;\mathrm{off}-\mathrm{dialysis}$$

This approach yielded an estimate of total CL_HDF,urea_ of 310 ml/min, i.e. larger than the diffusive clearance mechanistically predicted from filter characteristics, blood flow, and dialysate flow (predicted urea clearance: 230 ml/min, **Table 1**) (Gotta et al., 2020). The individual urea generation rate was estimated to 0.056 mmol/min.

The complete NONMEM model codes for ganciclovir and urea can be found in the supplemental data. All model predictions are shown in **Figure 1**.

### Review of the Literature

Publications relating to intermittent renal replacement therapy for comparison of our data were searched on MEDLINE during October 2019 using “renal dialysis” (MeSH) AND (“ganciclovir” OR “valganciclovir”). This search resulted in 32 references, whose abstracts were screened for pharmacokinetic data, which were then extracted from the full publication. In total seven publications reporting pharmacokinetic data in patients on intermittent hemodialysis were identified. Reported and calculated pharmacokinetic parameters of interest are summarized in **Table 3**.

**Table 3:** Review of existing literature (chronologically ordered) on ganciclovir elimination during different types of intermittent renal replacement therapy (RRT) and comparison with estimates for the present patient.

Reference (number of patients)	RRT type (duration)	Filter	Prescription	Clearance (ml/min)	ER/RR
Lake et al., 1988 (n = 1)	HD (4 h)	Cordis Dow 4000 Low UF	Q_B_ = 250 ml/min Q_D_: -	CL_D_ = 68.5 per 1.76 m^2^ CL_R,off_ = 10.1 per 1.76 m^2^ CL_tot,on_= 78.6 per 1.76 m^2^	ER = 0.29 RR: 50%
Sommadossi et al., 2019 (n = 2)	HD (4 h)	–	Q_B_ = 300 ml/min Q_D_ = 800 ml/min	–	ER: - RR = 53 ± 11% (up to 70%)
Swan et al., 1991 (n = 1)	HD (4 h)	Lundia IC 3L Gambro, 0.8 m^2^	Q_B_ = 250 ml/min Q_D_: -	CL_D_ = 48.3^*1^ CL_R,off_ = 35.5	ER = 0.29 RR = 62%
Combarnous et al., 1994 (n = 1)	HD (5 h)	high permeability dialyzer, 1.1 m2	Q_B_ = 200 ml/min Q_D_ = 500 ml/min	CL_D_: NR CL_R,off_ = 2.6	ER: - RR = 52%
Pescovitz et al., 1998 (n = 6)	HD (4 h)	–	–	–	ER: - RR = 53%
Czock et al., 2002 Czock and Rasche, 2004 (n = 6)	HD (4 h)	–	–	CL_D_ = 93.3 CL_R,off_ = 7.7 CL_tot,on_ = 106	ER: - RR ≈ 67% (visually) (Czock et al., 2002)/≈49–58% (Czock and Rasche, 2004),^*2^
Czock 2004	HD (4 h)	–	–	–	
Present case (n = 1)	HDF (4.4–4.7 h)	FX CorDiax 100, Fresenius (2.2 m2, KoAurea: 1545 ml/min)	Q_B_ = 350 ml/min Q_D_ = 525 ml/min UF: 34–40 L/session	CL_D_ = 445 CL_R,off_ = 29 CL_tot,on_ = 474	ER: 0.62 (±0.20) RR: ≥90%

RR, reduction ratio, calculated as 1-C_end_/C_start_ = (C_start_− C_end_)/C_start_ × 100%; ER, extraction ratio, calculated as (C_pre-filter_ – C_post-filter_)/C_pre-filter_; Q_B_, blood flow (ml/min); Q_D_, dialysate flow (ml/min); UF, ultrafiltration volume; CL_D_, ganciclovir dialysis clearance reported or calculated; CL_R,off_, residual renal ganciclovir clearance off-dialysis; CL_tot,on_, total ganciclovir clearance during dialysis (=CL_D_ + CL_R,off_). ^*1^Note that distribution exclusively to plasma was assumed in the calculation. ^*2^Assuming this value to approximate the fraction of dose removed from the body, the lower estimate accounts for rebound.

## Discussion

We present a case of an inadvertent eight-fold ganciclovir overdose in a 70-year-old kidney transplant patient, successfully treated with two sessions of intermittent HDF. Retrospective analysis of ganciclovir pharmacokinetic data revealed that HDF was much more efficient at removing ganciclovir from the patient’s plasma (reduction ratio: ≥90%) than previously reported for endstage-renal disease patients on chronic HD (50–67%, **Table 3**).

While it must be noted that residual renal ganciclovir clearance in our patient (estimated to be 30 ml/min by Bayesian feedback, i.e. twice her creatinine clearance) may have contributed to the high reduction ratio during a HDF session of 4.4 h, model-based analysis showed that it contributed to only 7% of total clearance during HDF (estimated to be 475 ml/min). A model-based analysis was considered necessary to calculate CL_HDF_ due to the delayed two-compartmental distribution kinetics of the drug (Czock et al., 2002; Caldés et al., 2009) and since assessment of dialysis clearance by the ER only is complicated by limited knowledge about the distribution into blood cells that would be required to make correct assumptions about the effective pharmacokinetic plasma flow through the filter (Atkinson and Umans, 2009). The ganciclovir CL_HDF_ estimate of 445 ml/min was very close to the sum of prescribed blood flow and ultrafiltration rate (350 + 131 ml/min = 481 ml/min) and was higher than urea dialysis clearance estimated (310 ml/min). This suggests an accumulation of ganciclovir in the blood cells in this patient (R_bp_ > 1). To our knowledge, R_bp_ has not been measured *in vivo* for ganciclovir. Based on its pharmacologic action, requiring intracellular phosphorylation leading to a long intra-cellular half-life (discussed in the introduction) and accumulation in peripheral blood mononuclear cells (Billat et al., 2016), it may be hypothesized that an R_bp_ > 1 would pharmacologically be expected. An erythrocyte/plasma value of 1.11 has indeed been measured *in vitro* by Perrottet et al. (2007). We acknowledge that an *in silico* estimate of 0.89 has been proposed (Lukacova et al., 2016)). in contrast to our expectation, which is however usually based on physiochemical properties only (e.g. polarity and lipophilicity) (Peters, 2012) and which may ignore active transmembrane transport mechanisms known for ganciclovir (Lukacova et al., 2016). If ganciclovir would distribute like the small molecule urea (molecular weight: 60 Da) homogenously and passively between plasma and red blood cells (R_bp_ = 1), CL_HDF_ of ganciclovir could physiologically not exceed the estimated CL_HDF,urea_ value of 310 ml/min. Collection of dialysate and ultrafiltrate to measure the recovered ganciclovir amount and CL_HDF_ directly would have been additionally useful as gold-standard method, but was not practical. Serial measurements of intra-cellular ganciclovir and application of a multi-compartment distribution model (Billat et al., 2016) could have further enhanced our mechanistic understanding of the high CL_HDF_ calculated. Importantly, highly efficient elimination of ganciclovir by HDF as reported here may not be sufficient to prevent hematologic and neurologic toxicity completely as intracellular accumulation still may occur. Our patient did not show any signs of neurotoxicity and she recovered from intermittent leukopenia, which was apparent before overdosing and may have other reasons as cytotoxicity due to CMV infection.

Interestingly however, there was a very strong correlation between the ganciclovir and urea reduction ratio calculated (both ratios calculated to 92% during the first session), which may be an approximation for the amount of drug removed during HDF. This may suggest—independent of red blood cell distribution—that efficiency of urea removal can be used as a surrogate marker for ganciclovir removal. The estimated CL_HDF,urea_ of 310 ml/min exceeded the mechanistically predicted diffusive *in vivo* urea clearance of 230 ml/min by 35%, suggesting significant contribution of convective clearance by the high ultrafiltration used in this HDF setting. The high blood flow compared to cases reported in the literature probably also contributed to a high diffusive clearance. The difference of 80 ml/min, relating to convective urea clearance, corresponds to 0.61–0.67 of the ultrafiltration rate used. This fraction may be interpreted as net sieving coefficient, which is expected to be reduced in the presence of diffusion (Tattersall and Blankestijn, 2019) compared to its expected value of 1.

In conclusion, this case illustrates highly efficient elimination of ganciclovir by intermittent HDF in a patient treated for inadvertent overdose potentially associated with intermittent leukopenia. Ganciclovir elimination by targeted intense HDF largely exceeded elimination reported by previous publications for patients on intermittent HD. The high elimination in the present case may be explained by several factors. First, a relevant contribution of convection to CL_HDF_ at the high ultrafiltration rate used could be quantified for the reference molecule urea. Second, the blood flow of 350 ml/min was higher and the HDF session duration of 4.4–4.7 h was longer than in previous references reporting on ganciclovir elimination during HD. Third, while it is difficult to conclude on the importance of filter characteristics, since details were frequently not provided in the literature, the filter used in this case may have been characterized by a larger surface area and higher dialytic efficiency than filters used in the past. In fact, some drug dose recommendations for patients on RRT issued before the year 2000 are likely outdated due to advanced technology (Mueller and Smoyer, 2009). The reduction ratio of reference molecules like urea, which is routinely assessed during HD and HDF, could be evaluated as an indicator of ganciclovir removal in future studies.

## Data Availability Statement

All datasets presented in this study are included in the article/**Supplementary Material**.

## Ethics Statement

Written informed consent was obtained from the individual for the publication of any potentially identifiable images or data included in this article.

## Author Contributions

Conception/data acquisition: MG, AJ. Conception/data analysis: DM, MP, VG, and AL-T. All authors were involved in interpretation of data. VG and AJ drafted the manuscript. All authors contributed to the article and approved the submitted version.

## Conflict of Interest

The authors declare that the research was conducted in the absence of any commercial or financial relationships that could be construed as a potential conflict of interest.
