# Supplementary material for: Intensive Hemodiafiltration Successfully Removes Ganciclovir Overdose and Largely Exceeds Reported Elimination During Hemodialysis—A Case Report and Review of the Literature
Source: Front Pharmacol. 2020 Jun 12;11:882. doi: 10.3389/fphar.2020.00882 (PMC7303306; doi:10.3389/fphar.2020.00882)
Supplement: Supplementary file 1 [file DataSheet_1.docx]

## Ganciclovir kinetic model (NONMEM model code)

$PROBLEM PK

$INPUT ID AMT TINF RATE DV EVID CMT TIME HDF OCC

; ID: individual identifier (=1 for all)

; AMT: amount of ganciclovir (mg)

; TINF: infusion time

; RATE: rate of infusion

; DV: ganciclovir concentration (mg/L)

; EVID: event identification (0=dose, 1=observation, 2=prediction at unobserved time point)

; CMT: compartment (1=prefilter and intra-dialytic samples, 3=postfilter samples)

; TIME since first ganciclovir dose (h)

; HDF: indicator variable (on HDF=1, off HDF=0)

; OCC: occasion (1= 1st HDF session, 2=2nd HDF session)

$DATA data_ganciclovir.csv

IGNORE=@

$SUBROUTINES ADVAN6 TRANS1 TOL=5

$MODEL

COMP=(CENTRAL, DEFDOSE)

COMP=(PERIPH)

COMP=(POSTFILTER)

COMP=(HDF)

COMP=(URINE)

$PK

; ----- inter-session variability for QEFF / ER respectively ----

OCC1 = 1

IF(OCC.EQ.2) OCC1 = 0

OCC2 = 0

IF(OCC.EQ.2) OCC2 = 1

IOV_CLD = OCC1*ETA(3) + OCC2*ETA(4)

; ----------- patient characteristics --------------

CL_CRT = 14 ; estimated creatinine clearance (mL/min) according to Cockroft-Gault

; ----------- typical pharmacokinetic parameters -------

TVCL = THETA(4) * (CL_CRT/57)

TVV1 = THETA(1)

V2 = THETA(2) * 44/66

Q = THETA(3) * (44/66)**0.75

; ----------- individual pharmacokinetic parameters -------

V1 = TVV1 * EXP(ETA(1))

CL = TVCL * EXP(ETA(2))

CLD = THETA(7)

QEFF = THETA(8) * EXP(IOV_CLD)

; ----- calculated/transformed kinetic parameters -------------

K12 = Q/V1

K21 = Q/V2

K10 = CL/V1

KD = CLD/V1

S1 = V1

; ------ differential equation --------------

$DES

DADT(1) = - K12*A(1) + K21*A(2) - K10*A(1) - KD*HDF*A(1)

DADT(2) = K12*A(1) - K21*A(2)

DADT(4) = KD*HD*A(1)

DADT(5) = K10*A(1)

; ------ error model and post-filter prediction --------

$ERROR

CP = A(1)/V1

COUT = CP*(1-CLD/QEFF)

; amounts in different compartments

ACP = A(1)

APERI = A(2)

AHDF = A(4)

AURINE = A(5)

IPRED = CP

W = SQRT(THETA(5)**2*IPRED**2 + THETA(6)**2)

Y = IPRED + W*EPS(1)

IRES = DV-IPRED

IWRES = IRES/W

IF(CMT.EQ.3) THEN

IPRED = COUT

Y = COUT * EXP(ERR(2))

IRES = DV-IPRED

ENDIF

; ------ fixed effect parameters ---------------------

$THETA

; values according to Caldes et al. 2009

31.9 FIX ; V1

32.0 FIX ; V2

10.2 FIX ; Q

7.49 FIX ; CL

0.143 FIX ; Prop.RE (sd)

0.465 FIX ; Add.RE (sd)

(0, 7) ; initial estimate of CLD

(0, 21) ; initial estimate of QEFF

; ------ random effect parameters ---------------

$OMEGA

; inter-individual variability (Caldes et al. 2009)

0.227 FIX ; IIV V1

0.107 FIX ; IIV CL

; inter-session variability, initial estimate

$OMEGA BLOCK(1) 0.1

$OMEGA BLOCK(1) SAME

$SIGMA

1 FIX ; Pre-filter & Intra-dialytic samples

0.1 ; proportional error of post-filter samples

$EST METHOD=1 INTER MAXEVAL=9000 NOABORT SIG=3 PRINT=1 POSTHOC

$COV

$TABLE ID TIME DV MDV EVID CMT IPRED COUT CP IWRES HD ACP APERI AHDF AURINE ONEHEADER NOPRINT FILE=sdtab0024

$TABLE TVCL TVV1 CL V1 V2 Q CLD QEFF FIRSTONLY ONEHEADER NOPRINT FILE=patab0024

## Urea kinetic model (NONMEM model code)

$PROBLEM PK

$INPUT ID DV EVID CMT TIME HDF

; ID: individual identifier (=1 for all)

; DV: observation in mmol/L

; EVID: event identification (1=observation, 2=prediction at unobserved time point)

; CMT: compartment

; TIME since first ganciclovir dose (h)

; HDF: indicator variable (on HDF=1, off HDF=0)

$DATA data_nm_urea2.csv

IGNORE=@

$SUBROUTINES ADVAN6 TRANS1 TOL=5

$MODEL

COMP=(CENTRAL)

COMP=(PERIPH)

$PK

; ----------- patient characteristics --------------

WEIGHT = 44 ; weight (kg)

CL_CRT = 14 ; estimated creatinine clearance (mL/min)

BL = THETA(2) ; baseline = pre-HDF measured urea concentration (21 mmol/L)

; baseline estimation better than fixing it to measured valued according to Dansirikul 2008

; ------- urea distribution parameters (Gotta et al.) -----------------

Q_Lmin = 0.585 ; intercompartmental clearance (L/min), scaled based on weight (correlated with cardiac output)

fVtot = 0.59 ; urea volume of distribution (L/kg) = total body water fraction (Watson prediction assuming height = 160 cm)

fV1 = 0.36 ; fraction of central urea volume

Q = Q_Lmin * 60 ; L/min * (60 min/h) = L/h

; ------- urea generation rate -----------------

KIN = 0.17 * 60 * EXP(ETA(3)) ; mmol/min -> mmol/h (Pfister et al: mean: 0.17 mmol/min, interindividual variability: 39%)

; ------ urea clearance --------------------------------

CLD = THETA(1) * 60/1000 ; HDF clearance (mL/min) -> L/h

CLR = CL_CRT * 60/1000 * 0.85/1.15 ; renal clearance (mL/min) -> L/h (proportional to creatinine clearance by factor 0.85/1.15)

CL = CLR + CLD*HDF ; total clearance (L/h)

; ----- calculated/transformed kinetic parameters -------------

Vtot = fVtot * WEIGHT ; L

V1 = Vtot * fV1 ; L

V2 = Vtot * (1-fV1) ; L

K12 = Q/V1 ; 1/h = L/h * (1/L)

K21 = Q/V2 ; 1/h = L/h * (1/L)

KR = CLR/V1 ; 1/h = L/h * (1/L)

KD = CLD/V1 ; 1/h = L/h * (1/L)

S1 = V1

; ------ initial conditions for amount in central and peripheral compartment

A_0(1) = BL * V1

A_0(2) = BL * V2

; ------ differential equation --------------

$DES

DADT(1) = - K12*A(1) + K21*A(2) - KR*A(1) - KD*HDF*A(1)

DADT(2) = KIN + K12*A(1) - K21*A(2)

; ------ error model (Gotta et al.) -----------------------

$ERROR

IPRED = F

W = SQRT(THETA(3)**2*IPRED**2 + THETA(4)**2)

Y = IPRED + W*EPS(1)

IRES = DV-IPRED

IWRES = IRES/W

; ------ fixed effect parameters ---------------------

$THETA

(0, 230) ; initial estimate of CLD

(0, 20) ; initial estimate of BL

0.108 FIX ; proportional error: 10.8%

0 FIX ; no additive error (= 0)

; ------ random effect parameters ---------------

$OMEGA 0.15 FIX ; variance of prior inter-individual variability in KIN (CV = 38%)

$SIGMA

1 FIX ; Proportional error PK

$EST METHOD=1 INTER MAXEVAL=9000 NOABORT SIG=3 PRINT=1 POSTHOC

$COV

$TABLE ID TIME DV MDV EVID IPRED IWRES PREPOST HD ONEHEADER NOPRINT FILE=sdtab0015

$TABLE CL V1 V2 Q CLD CLR KIN FIRSTONLY ONEHEADER NOPRINT FILE=patab0015
